# Role of Enteric Glia as Bridging Element between Gut Inflammation and Visceral Pain Consolidation during Acute Colitis in Rats

**DOI:** 10.3390/biomedicines9111671

**Published:** 2021-11-12

**Authors:** Elena Lucarini, Luisa Seguella, Martina Vincenzi, Carmen Parisio, Laura Micheli, Alessandra Toti, Chiara Corpetti, Alessandro Del Re, Silvia Squillace, Daniela Maftei, Roberta Lattanzi, Carla Ghelardini, Lorenzo Di Cesare Mannelli, Giuseppe Esposito

**Affiliations:** 1 Department of Neuroscience, Psychology, Drug Research and Child Health, Neurofarba, Pharmacology and Toxicology Section, University of Florence, 50139 Florence, Italy; elena.lucarini@unifi.it (E.L.); carmen.parisio@unifi.it (C.P.); laura.micheli@unifi.it (L.M.); alessandra.toti@unifi.it (A.T.); carla.ghelardini@unifi.it (C.G.); 2 Department of Physiology and Pharmacology “V. Erspamer”, Sapienza University of Rome, 00185 Rome, Italy; seguella@msu.edu (L.S.); martina.vincenzi@uniroma1.it (M.V.); chiara.corpetti@uniroma1.it (C.C.); alessandro.delre@uniroma1.it (A.D.R.); daniela.maftei@uniroma1.it (D.M.); roberta.lattanzi@uniroma1.it (R.L.); giuseppe.esposito@uniroma1.it (G.E.); 3 Department of Physiology, Michigan State University, East Lansing, MI 48824, USA; 4 Department of Pharmacology and Physiology and the Henry and Amelia Nasrallah Center for Neuroscience, Saint Louis University School of Medicine, St. Louis, MO 63104, USA; silvia.squillace.1@health.slu.edu

**Keywords:** inflammatory bowel disease, S100β, TRPV1 receptors, enteric glia, astrocytes, myenteric plexus, periaqueductal grey matter, dorsal root ganglion

## Abstract

Acute inflammation is particularly relevant in the pathogenesis of visceral hypersensitivity associated with inflammatory bowel diseases. Glia within the enteric nervous system, as well as within the central nervous system, contributes to neuroplasticity during inflammation, but whether enteric glia has the potential to modify visceral sensitivity following colitis is still unknown. This work aimed to investigate the occurrence of changes in the neuron–glial networks controlling visceral perception along the gut–brain axis during colitis, and to assess the effects of peripheral glial manipulation. Enteric glia activity was altered by the poison fluorocitrate (FC; 10 µmol kg^−1^ i.p.) before inducing colitis in animals (2,4-dinitrobenzenesulfonic acid, DNBS; 30 mg in 0.25 mL EtOH 50%), and visceral sensitivity, colon damage, and glia activation along the pain pathway were studied. FC injection significantly reduced the visceral hyperalgesia, the histological damage, and the immune activation caused by DNBS. Intestinal inflammation is associated with a parallel overexpression of TRPV1 and S100β along the gut–brain axis (colonic myenteric plexuses, dorsal root ganglion, and periaqueductal grey area). This effect was prevented by FC. Peripheral glia activity modulation emerges as a promising strategy for counteracting visceral pain induced by colitis.

## 1. Introduction

Visceral hyperalgesia is one of the most common gastrointestinal issue contributing to discomfort and impaired quality of life in patients with functional gastrointestinal disorders and inflammatory bowel diseases (IBDs) [1]. Current theories suggest that functional remodeling of neural networks controlling visceral perception and immune activation contribute to the development of abdominal pain during colitis which persists long after acute inflammation has resolved. In this context, the release of proinflammatory mediators by macrophages and other immune cells infiltrating the intestinal mucosa concurs to drive neuroplastic changes [2], such as the increase in transient receptor potential vanilloid receptor type-1 (TRPV1) on nociceptors innervating the intestine, contributing to the development of visceral hyperalgesia [3,4]. Although little is still known about the mechanisms that drive these alterations, it is increasingly clear that glia is another cell type involved in peripheral sensitization and intestinal inflammation. Glia present at all sites along pathways that convey nociceptive information deriving from the intestine, including astrocytes, microglia, and satellite cells in the dorsal root ganglia (DRGs), have the potential to influence visceral sensation [5,6,7]. As a unique class of peripheral glia of the enteric nervous system (ENS), enteric glia responds to pathophysiological perturbations of the enteric environment to protect the nervous system from damage and maintain local homeostasis [8]. However, enteric glial activity undergoes extensive changes during the intestinal inflammation that have the potential to alter the function of surrounding neural and non-neuronal cells and contribute to neuroinflammation and neurodegeneration associated with colitis [9,10,11]. Reactive enteric glia exhibits a pro-inflammatory phenotype and overexpression of the enteric glia-derived Ca^+2^/Zn^+2^-binding protein S100β has been linked to the onset and maintenance of intestinal inflammation in human colon [12]. This neurotrophin promotes mucosal macrophage recruitment and enhances inflammation, by orchestrating a wide range of signaling activation pathways that are directly correlated to the severity of intestinal degenerative processes [11,13]. Although the role of enteric glia in mediating the intestinal inflammation and transmitting glia-to-glia signals via the gut–brain axis has been extensively documented [11,14,15], whether these cells might influence visceral hypersensitivity is still relatively unknown. We addressed this hypothesis by altering glial activity by intraperitoneal administration of fluorocitrate (FC) in a model of colitis induced by 2,4-dinitrobenzenesulfonic acid (DNBS) and studied its effects on (1) visceral sensitivity; (2) local immune responses; and (3) nociceptive pathway along gut–brain axis that extends from the colonic myenteric plexuses to the brain areas involved in the control of visceral perception.

## 2. Materials and Methods

### 2.1. Animals

**Figure 1:**
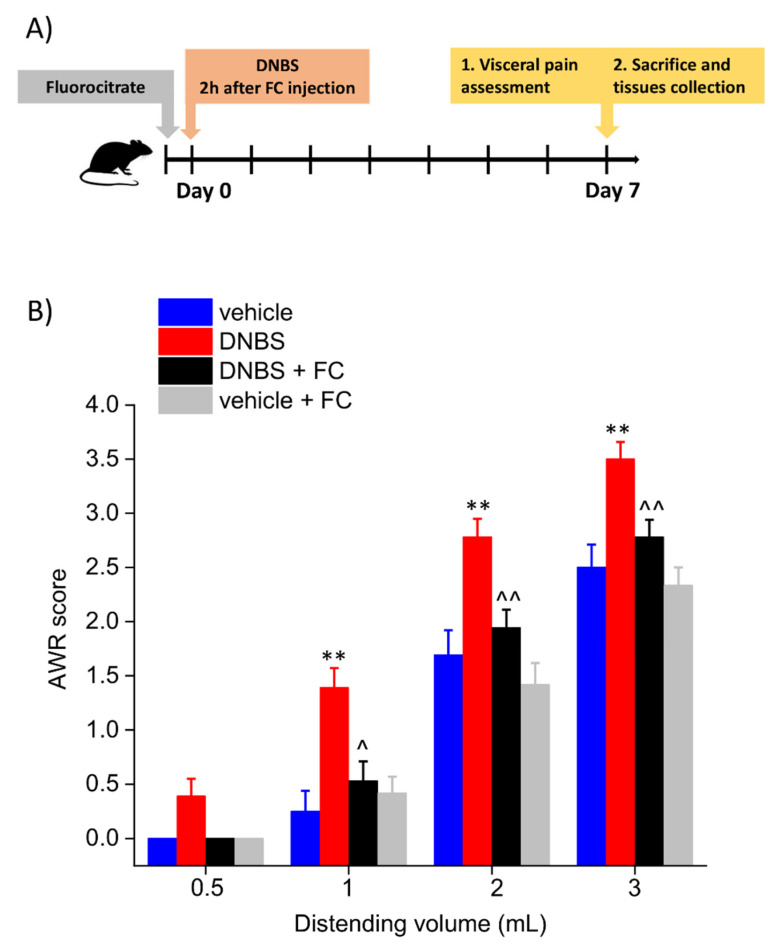
Effect of systemic fluorocitrate administration on the development of visceral pain after DNBS injection. Experimental scheme (**A**); Abdominal withdrawal reflex (AWR) in response to colorectal distension (**B**). Each value represents the mean ± SEM of 8 animals per group. ** *p* < 0.01 vs. vehicle. ^ *p* < 0.05 and ^^ *p* < 0.01 vs. DNBS.

Three-month-old male Sprague-Dawley rats (Envigo, Varese, Italy) with an initial average weight of 220–250 g were used for the experiments. Four animals per cage (size 26 × 41 cm) were housed in CeSAL (Centro Stabulazione Animali da Laboratorio, University of Florence) and used at least 1 week after their arrival. All animals were maintained on a 12-h light/dark cycle in a temperature-controlled environment (23 ± 1 °C) with access to food and water ad libitum. All animal manipulations were carried out according to the Directive 2010/63/EU of the European parliament and of the European Union council (22 September 2010) on the protection of animals used for scientific purposes. The ethical policy of the University of Florence complies with the Guide for the Care and Use of Laboratory Animals of the US National Institutes of Health (NIH Publication No. 85–23, revised 1996; University of Florence assurance number: A5278-01). Formal approval to conduct the described experiments was obtained from the Animal Subjects Review Board of the University of Florence (543/2017-PR). Experiments involving animals have been reported according to ARRIVE guidelines [16]. All efforts were made to minimize animal suffering and to reduce the number of animals used. Eight animals per each group were used in the experimental procedures, which are described in Figure 1A. The same animals were used for both the behavioural assessments and the histological analysis.

### 2.2. Induction of Colitis

Colitis was induced as previously described [17]. Briefly, animals were anesthetized lightly with isoflurane (2%) and 30 mg of 2,4-Dinitrobenzenesulfonic acid (DNBS; Sigma-Aldrich, Milan, Italy) in 0.25 mL of 50% ethanol was administered intrarectally (8 cm proximal to the anus) through a polyethylene PE-60 catheter. As a vehicle group, rats received 0.25 mL of saline solution.

### 2.3. Fluorocitrate Solution Preparation

FC solution was prepared as previously described [18,19]. Briefly, a clear solution of d,l-fluorocitric acid barium salt (Sigma-Aldrich, Milan, Italy) was obtained by dissolving the salt in 1 mM of hydrochloric acid. Then, barium was separated from the solution as barium sulphate by adding a slight excess of sodium sulphate. This solution was filtered through a 0.2-μm filter, and distilled water was added to make up the final volume. NaCl was added to make the solution isotonic before use. The concentration was adjusted to have a final solution of fluorocitrate at 10 μmol mL^−1^ [20,21]. Enteric glial function was perturbed in vivo with a single injection (IP) of FC (10 µmol Kg^−1^) 2 h before inducing colitis, according to a slightly modified reported method for enteric glia [22] and central glia [23]. Behavioural assessments and histological analysis were performed 7 days after DNBS and FC injection in the animals, as reported in the experimental scheme (Figure 1A). This concentration does not cause intestinal inflammation or neurodegeneration, and no drug toxicity has been reported by prior studies that used this dose to impair glial function in the myenteric plexus [21,23].

### 2.4. Assessment of Visceral Sensitivity by Abdominal Withdrawal Reflex to Colorectal Distension

To perform colorectal distension, a balloon (length: 4.5 cm) tightened to an embolectomy catheter and connected with a syringe was inserted into the colon of animals undergoing a light anaesthesia (2% isoflurane). The external part of the catheter was then fixed to the tail. The animals were allowed to recover from anaesthesia for 30 min before starting the tests. During the measure, the balloon was filled with increasing volumes of water (0.5, 1, 2, 3 mL; 5 min was the time elapsed between two consecutive distensions). Visceral sensitivity to colorectal distension were assessed by assigning a semi-quantitative score (0 to 4) to the abdominal withdrawal reflex (AWR) of animals, as described previously [17,24]: no behavioural response to colorectal distention (0); immobility during colorectal distention and occasional head clinching at stimulus onset (1); mild contraction of the abdominal muscles but absence of abdomen lifting from the platform (2); observed strong contraction of the abdominal muscles and lifting of the abdomen off the platform (3); and arching of the body and lifting of the pelvic structures and scrotum (4).

### 2.5. Histological Analysis of Colon

The evaluation of colonic macroscopic damage was performed in accordance with the criteria and score reported previously [25]: presence of adhesions between colon and other intra-abdominal organs (0–2); consistency of colonic faecal material (0–2); thickening of colonic wall (expressed in mm); presence and extension of hyperaemia and macroscopic mucosal damage (0–5). Colon segment (around 1 cm) was fixed in 4% paraformaldehyde for 24 h, dehydrated in alcohol, included in paraffin, and finally cut into 5-μm sections. Descriptive evaluations were carried out on haematoxylin–eosin-stained sections of full-thickness samples obtained from the distal colon. Mast cells (MCs) and eosinophils were detected by histochemical GIEMSA staining (Sigma-Aldrich, Milan, Italy). MCs were also labelled by immunoenzymatic histochemistry, using a rabbit mast cell tryptase antibody (GeneTex, Irvine, CA, USA) followed by a DAB staining (Sigma-Aldrich, Italy) [26]. Foam cells abd macrophage cells (containing lipid droplets and hemosiderin) were revealed by the natural yellow-brown staining of granules [27,28,29]. Digitalized images were collected at 40X magnification by a Leica DMRB light microscope, equipped with a DFC480 digital camera (Leica Microsystems, Milton Keynes, UK), and analysed quantitatively using the ImageJ software. Two blind investigators evaluated independently the cellular density (cell number/respective arbitrary field). At least five independent arbitrary optical fields (0.1 mm^2^) collected from the submucosa of each animal were analysed.

### 2.6. Circular Muscle Myenteric Plexus (CMMP) Whole-Mount Preparation

Segments of colon were immediately removed from euthanized animals and placed in ice-cold DMEM/Ham’s F-12 nutrient mixture (Corning, Kaiserslautern, Germany). The full-thickness tissues were then opened along the mesenteric border and pinned flat with mucosa facing-up in Sylgard-coated Petri dishes (Dow Italia s.r.l., Milan, Italy). The mucosa was removed by cutting at the level of the lamina propria while separating the layers with forceps. Tissues were then flipped, and the longitudinal muscle and serosa were removed by microdissection to obtain live whole mounts with intact myenteric plexus lying atop of the preparation (CMMP).

### 2.7. Immunofluorescence

DRG and brains were dissected from rats 7 days after DNBS injection and were fixed overnight in ice-cold 4% PFA, rinsed in 1X PBS for 30 min, and then transferred to a 1X PBS solution containing 20% sucrose at 4 °C for 48 h. Tissues were mounted in OCT (optimal cutting temperature) compound, snap frozen in dry ice, and cut on a cryostat in 20-μm slices. All slices were collected onto SuperFrost Plus slides and air-dried at room temperature in the dark overnight before being immunolabeled. Colonic CMMP preparations were fixed overnight in Zamboni’s solution at 4 °C followed by a 30-min rinse in 1X PBS. Tissues were rinsed three times (10 min each) in PBS containing 0.1% Triton X-100 (T-PBS) followed by a 1 h incubation in blocking solution (containing 4% normal donkey serum, 0.1% Triton X-100, and 1% bovine serum albumin in 1X PBS) at room temperature. After three washes in T-PBS (10 min each), primary antibodies diluted in blocking solution were applied overnight at +4 °C (Table 1). Tissues were rinsed three times (10 min each) with T-PBS after removing the primary antibodies, and then secondary antibodies were applied for 2 h at room temperature (Table 1). Tissues were rinsed two times (10 min each) in 1X PBS, once in 0.1 M of phosphate buffer (10 min), and mounted in Fluoromount (SouthernBiotech, Birmingham, AL, USA). Antibody specificity was confirmed by pre-adsorption with the corresponding control peptides. Images were acquired through the 10×, 20×, and 40× (numerical aperture 0.25, 0.50, and 0.75, respectively) using a high-resolution digital camera (Nikon Digital Sight DS-U1) on an inverted Nikon Eclipse 80i microscope (Nikon corporation, Minato, Tokyo, Japan) and analysed offline using FIJI software (National Institutes of Health, Bethesda, MD, USA) and Imaris 9.5.1 (Bitplane Inc., South Windsor, CT, USA) to segment and overlay pictures. Final images were transferred to Adobe Photoshop CS6 (Adobe Adobe Incorporated, San Jose, CA, Stati Uniti) for the construction of figure sets. Acquisition parameters were optimized to each tissue as the fluorescence intensity of each probe differed between tissues. Thus, results were expressed as a percentage of PLP1 immunopositive cells that express S100β per area unit (μm^2^) in the given tissue without comparing expression between tissues. This percentage was compared to the expression of TRPV1 within the same tissue. Fluorescence intensity profiles for PLP1, S100β, and TRPV1 were measured by FIJI software as maximum grey values within the region of interest [arbitrary units (a.u.; μm^2^)].

### 2.8. Statistics

All measurements were made by researchers blinded to animal treatments. Data were analysed using “Origin 9” software (OriginLab, Northampton, MA, USA) by one or two-way analysis of variance (ANOVA) with a Bonferroni post-test, with *p* < 0.05 or 0.01 considered statistically significant, respectively. Results are shown as means ± standard error of the mean (SEM) of *n* assessments depending on the experiment.

## 3. Results

### 3.1. Enteric Glia Inhibition by FC Prevented the Development of Colitis-Related Visceral Hyperalgesia in Rats

The experimental scheme is reported in Figure 1A. Visceral pain was assessed in the animals by assigning a score to their abdominal withdrawal reflex (AWR) to colorectal distension (CRD), which was performed by progressively increase balloon volumes as a pressure stimulus to the colon (highest volume: 3 mL, to avoid tissue damage). Seven days after colitis induction, the AWR to 1–3 mL distending volume was significantly higher in DNBS-treated animals than in controls (vehicle; Figure 1B). Pre-treatment with FC effectively prevented the development of visceral hypersensitivity induced by DNBS in rats. Indeed, AWR to CRD (1–3 mL), resulted significantly lower in the DNBS group pre-treated with FC (DNBS + FC) than rats receiving vehicle (DNBS). By contrast, the intraperitoneal injection of FC did not alter the visceral sensitivity of control rats (vehicle + FC; Figure 1B).

### 3.2. Enteric Glia Inhibition by FC Reduced the DNBS-Induced Intestinal Damage in Rats

**Figure 2:**
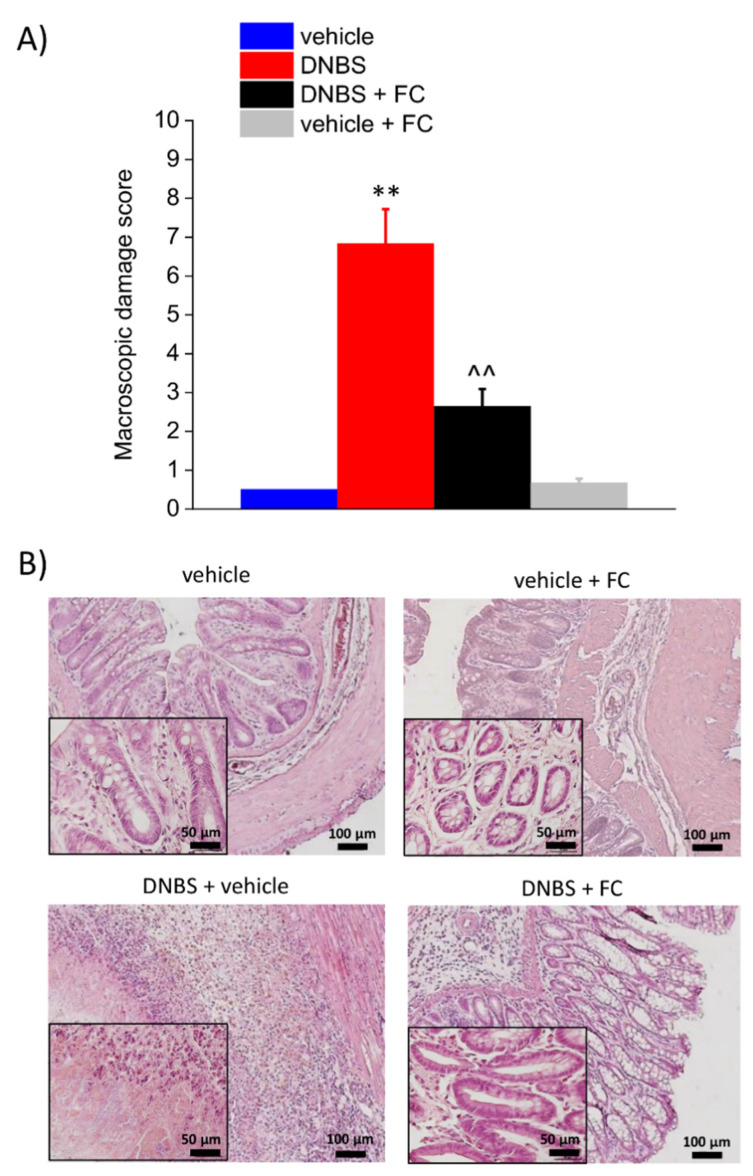
Effect of systemic fluorocitrate administration on colon damage induced by DNBS. (**A**) Colon macroscopic damage score. (**B**) Representative pictures of haematoxylin–eosin-stained sections of full-thickness colon. Original magnification: 10× and 40×. Each value represents the mean ± SEM of 8 animals per group. ** *p* < 0.01 vs. vehicle. ^^ *p* < 0.01 vs. DNBS. Original magnification: 10× and 40×. Scale bars: 50 and 100 μm.

On day 7, the macroscopic damage score was significantly higher in the DNBS group than in the vehicle group. FC administration effectively prevented the intestinal damage after DNBS injection in rats (Figure 2A). From a microscopic point of view, 7 days after DNBS injection, colonic samples showed a considerable mucosal injury with multiple ulcers and areas with lining epithelium loss, transmural immune cell infiltration, crypt abscesses, and oedema. In the group of animals receiving DNBS + FC, the tunica mucosa resulted mostly restored, though the crypts were elongated, and goblet cells displayed hypertrophy and atrophy depending on the zone. Moreover, despite the thickening of colonic walls, the presence of inflammatory infiltrate in the DNBS+FC group was significantly reduced and almost exclusively limited to the submucosa (Figure 2B). Control animals treated with FC (vehicle + FC) showed no macroscopic (Figure 2A) or microscopic signs of inflammation (Figure 2B), confirming that such tissue changes did not occur at the selected FC dosage.

### 3.3. Enteric Glia Inhibition by FC Reduced MCs Infiltration and Macrophages Activation in the Submucosa of DNBS Treated Rats, but Not Eosinophils Recruitment

**Figure 3:**
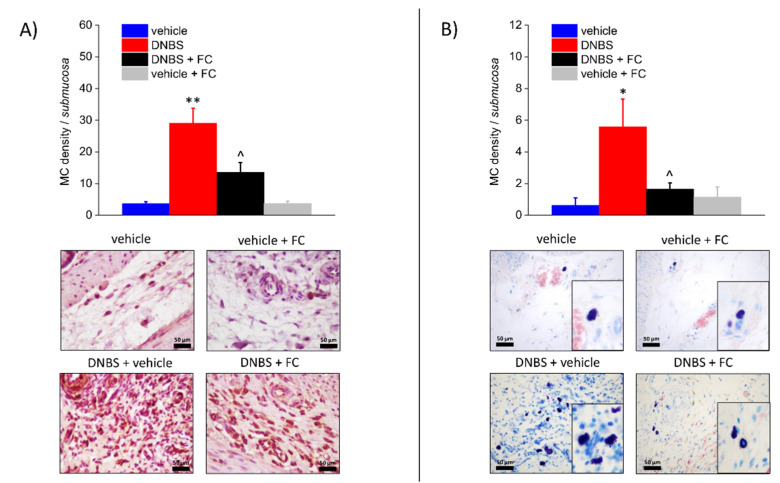
Effect of systemic fluorocitrate administration on submucosal MCs increase induced by DNBS. The panel shows pictures captured from submucosa of MCs stained with antibody to tryptase (**A**) or with GIEMSA (**B**). Column graphs display the mean values of MC density per area of colonic wall (cells/field) ± S.E.M. obtained from 6 animals for each group. * *p* < 0.05 and ** *p* < 0.01 vs. vehicle. ^ *p* < 0.05 vs. DNBS. Original magnification: 40×. Scale bar: 50 μm.

**Figure 4:**
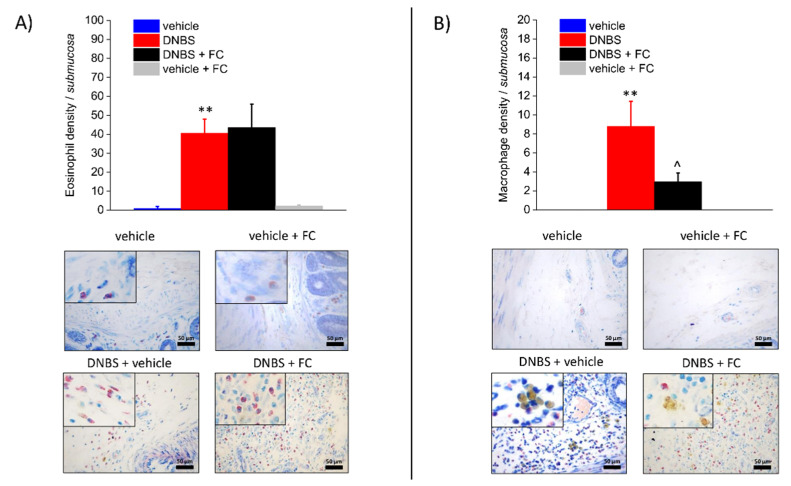
Effect of systemic fluorocitrate administration on submucosal eosinophils and activated macrophages increase induced by DNBS. The panel shows pictures captured from submucosa of eosinophils stained with GIEMSA (**A**) or foamy macrophages naturally stained by their yellow-brown granules (**B**). Column graphs display the mean values of eosinophil or macrophage density per area of colonic wall (cells/field) ± S.E.M. obtained from 6 animals for each group. ** *p* < 0.01 vs. vehicle. ^ *p* < 0.05 vs. DNBS. Original magnification: 40×. Scale bar: 50 μm.

Seven days after DNBS treatment, the colonic submucosa was associated with inflammatory cells infiltration, including MCs (Figure 3A,B), eosinophils (Figure 4A), and macrophages (Figure 4B). MCs, which were occasionally found along the submucosal colonic vessels in the control group, exhibited significantly increased density throughout the colonic wall of DNBS-treated rats, as assessed by tryptase detection with DAB (Figure 3A) and GIEMSA violet staining of MCs granules (Figure 3B). Eosinophil density was significantly augmented in the inflamed colonic submucosa in comparison with the eosinophils detected at submucosal level in the controls (Figure 4A). Further, activated macrophages markedly increased in the submucosa of DNBS-treated animals (Figure 4B). Administration of FC significantly decreased the number of MCs (Figure 3A,B) and activated macrophages (foam cells, Figure 4B). Otherwise, eosinophils infiltration was not influenced by FC in DNBS animals (Figure 4A). FC injection in control animals did not alter MCs, eosinophil, or macrophage density (Figure 3A,B and Figure 4A,B), confirming that FC does not affect the local immune response at the selected dosage.

### 3.4. DNBS Increases the Expression of S100β in the PLP1-Positive Glia and TRPV1 in the Colonic Myenteric Plexus

**Figure 5:**
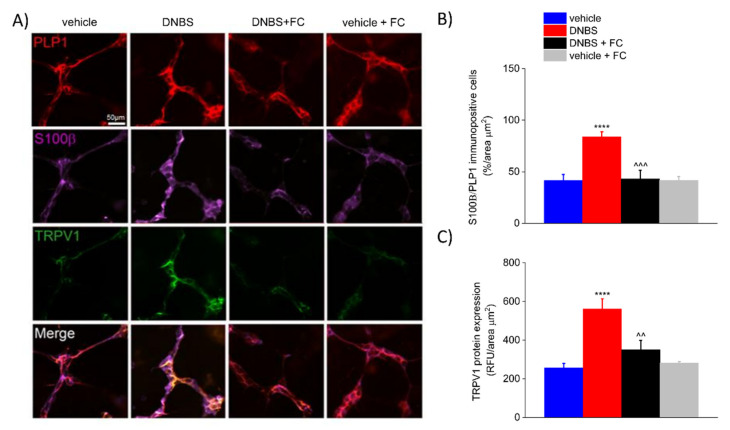
Effect of systemic fluorocitrate on S100β and TRPV1 increased expression within the colonic myenteric plexus of DNBS rats at day 7. (**A**) Immunofluorescence images show the expression of PLP1 (red), S100β (purple), and TRPV1 (green) in the myenteric plexus of the colon and (**B**,**C**) relative immunolabeling quantification at day 7 after colitis induction. Data were analysed by one-way ANOVA and Bonferroni post-hoc. Results are expressed as a mean ± SEM of the percentage of PLP1 immunopositive cells that co-express S100β per area unit (μm^2^) of n assessments. Results about TRPV1 expression are expressed as average relative fluorescence units (RFUs) ± SEM per area unit of (μm^2^) of n assessments. **** *p* < 0.0001 vs. vehicle, ^^^ *p* < 0.001 and ^^ *p* < 0.01 vs. DNBS. Original magnification: 20×. Scale bar: 50 μm.

Enteric glia constantly monitors and responds to changes in the surrounding environment to maintain local homeostasis [10,30,31,32]. Activation of enteric glia encompasses a wide range of changes in morphology and/or markers expression, such as GFAP, S100β, and receptors, as well as an increase or loss in physiological functions, with potentially deleterious effects on neighbouring cells [33,34,35]. Given that DNBS triggered an enteric glia-mediated inflammatory response in the colon and induced visceral hypersensitivity, we investigated what changes occurred in the colonic myenteric plexuses after colitis and whether FC antagonized these changes. Immunofluorescence analysis revealed an up-regulation of S100β in PLP1-positive cells (+102.11% vs. vehicle) and a parallel increase in TRPV1 (+119.0% vs. vehicle) within the myenteric plexus of DNBS rats compared with vehicle group (*p* < 0.0001, Figure 5A–C). This was significantly inhibited by FC treatment (*p* < 0.001 and *p* < 0.01, −48,58% in DNBS + FC vs. DNBS group for S100β expression in PLP1-positive cells; −37.66% in DNBS + FC vs. DNBS group for TRPV1 protein expression; Figure 5A–C), whereas no significant changes in S100β (+0.30% vs. vehicle) and TRPV1 (+9.89% vs. vehicle) protein expression were observed in vehicle rats treated with FC (Figure 5A–C). These results highlight that altered expression of neurotrophins by enteric glia correlates with neuroplastic changes in TRPV1 expression in the context of intestinal inflammation within enteric myenteric networks.

### 3.5. DNBS Elicited a S100β Increase in PLP1-Positive Cells and TRPV1 in the DRG

**Figure 6:**
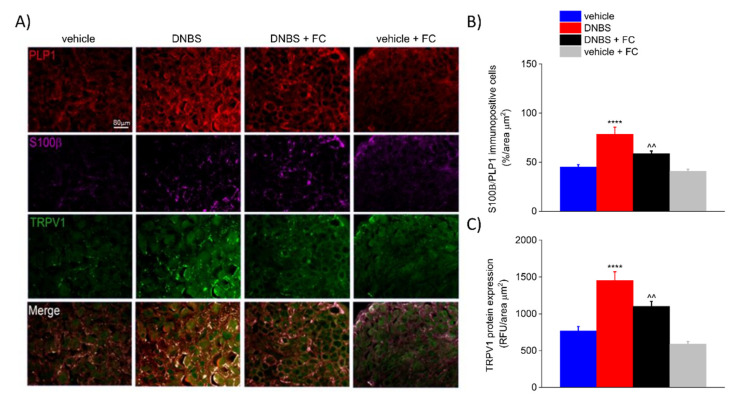
Effect of systemic fluorocitrate on S100β and TRPV1 increased expression in the DRG of DNBS rats at day 7. (**A**) Representative images show the expression of PLP1 (red), S100β (purple), and TRPV1 (green) in the DRG and (**B**,**C**) relative immunolabeling quantification at day 7 after colitis induction. Data were analysed by one-way ANOVA and Bonferroni post-hoc. Results are expressed as a mean ± SEM of the percentage of PLP1 immunopositive cells that express S100β per area unit (μm^2^) of n assessments. Results about TRPV1 expression are expressed as average relative fluorescence units (RFUs) ± SEM per area unit of (μm^2^) of n assessments. **** *p* < 0.0001 vs. vehicle and ^^ *p* < 0.01 vs. DNBS. Original magnification: 10×. Scale bar: 80 μm.

Increasing evidence suggests that astrocytes and microglia in the CNS and satellite glia in the DRG contribute to develop inflammatory-related pain states through persistent reactive gliosis and the release of neuromodulators [36,37]. This alters neuronal circuits involved in the transmission of pain signals in the nervous system and in pain perception [38,39,40]. To test whether DNBS-induced changes within the enteric neural circuits reverberate to the CNS, we performed the same immunohistochemical assessments carried out in the myenteric plexuses of the colon in the DRG (Figure 6). Immunofluorescence analysis revealed a significant increase in S100β (102.11% vs. vehicle) and TRPV1 (+89.45% vs. vehicle) in the DRG of DNBS-treated rats compared to the vehicle group on day 7 after colitis induction (*p* < 0.0001; Figure 6A–C). DNBS rats pre-treated with FC showed a significant decrease in S100β (−25.40% vs. DNBS) and TRPV1 (−24.12% vs. DNBS) expression (*p* < 0.001; Figure 6A–C). FC alone did no alter S100β (−9.14% vs. vehicle) and TRPV1 (−22.90% vs. vehicle) expression (Figure 6A–C). This confirms that FC prevented the glial response without inducing drug-related toxicity. These results mirror the scenario observed in the colonic myenteric plexuses, suggesting that DNBS-induced colitis triggers a parallel increase in S100β and TRPV1 within the neuronal networks ascending to the DRG.

### 3.6. DNBS Is Associated with Increased Expression of S100β and TRPV1 in the Periaqueductal Grey Area

**Figure 7:**
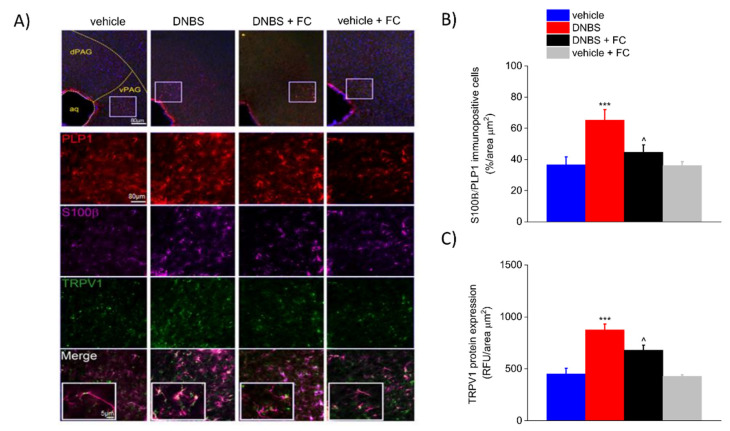
Effect of systemic fluorocitrate on S100β and TRPV1 increased expression in the periaqueductal grey area of DNBS rats at day 7 (**A**) Representative images show the expression of PLP1 (red), S100β (purple), and TRPV1 (green) in the periaqueductal grey area and (**B**,**C**) relative immunolabeling quantification at day 7 after colitis induction. Data were analysed by one-way ANOVA and Bonferroni post-hoc. Results are expressed as a mean ± SEM of the percentage of PLP1 immunopositive cells that express S100β per area unit (μm^2^) of n assessments. Results about TRPV1 expression are expressed as average relative fluorescence units (RFUs) ± SEM per area unit of (μm^2^) of n assessments. *** *p* < 0.001 vs. vehicle and ^ *p* < 0.1 vs. DNBS. Original magnification: 4×, 10×, and 40×. Scale bars: 5 and 40 μm.

Current theories suggest that widespread changes in the brain–gut axis contribute to the development of abdominal pain [36,39], although little is yet known about the mechanisms driving these changes along the neuron–glia networks. Given that our data show a glial-dependent increase in visceral hypersensitivity in DNBS rats, we hypothesised that DNBS-mediated changes observed in the colonic myenteric plexus and in the DRG could reverberate to the CNS and affect brain areas involved in pain perception. Similarly, the expression of S100β (+78.16% vs. vehicle) and TRPV1 (+94.07% vs. vehicle) was significantly increased in the periaqueductal grey area (PAG) of DNBS rats compared to vehicle group on day 7 after colitis induction (*p* < 0.001 Figure 7A–C). FC pre-treatment significantly inhibited this response, showing decreased expression of S100β (−31.61% vs. DNBS) and TRPV1 (−22.23% vs. DNBS) compared to DNBS group (*p* < 0.001; Figure 7A–C). Vehicle rats treated with FC showed no signs of reactive gliosis in the periaqueductal grey area (−1.37% in vehicle + FC vs. vehicle group for S100β expression in PLP1-positive cells; −4.71% in vehicle + FC vs. vehicle group for TRPV1 protein expression; Figure 7A–C), confirming that FC did not cause significant changes in the CNS.

## 4. Discussion

In the present study, we have shown that enteric glia is involved in the development of visceral hypersensitivity induced by DNBS in rats. Impairment of glial activity by a single administration of FC prevented intestinal damage, and the overexpression of S100β and TRPV1 along the pain signalling pathway, resulting in a reduction in DNBS-induced visceral hypersensitivity.

Abdominal pain is a common gastrointestinal issue in IBD patients, which is used as a patient-reported outcome to evaluate the efficacy of new therapies [41]. Current theories suggest that intestinal inflammation drives neuroplastic changes within the enteric circuits that alter neuronal sensitivity and neurotransmission. This leads to the development of visceral pain and intestinal dysmotility which often persist long after the resolution of acute inflammation [17,42,43,44]. In this context, the release of proinflammatory mediators by immune cells contributes to amplify the nociceptive signalling and to increase the intestinal damage [45]. Previous studies showed a marked infiltration of MCs within the mucosa and submucosa of IBD patients, which correlated with visceral hyperalgesia and worsening of clinical symptoms [46,47]. Similarly, in DNBS rats, we observed an increase in the number of MCs, along with enhanced eosinophils infiltration and macrophages activation, which corresponded to a higher macroscopic and histological damages. A single injection of FC into DNBS animals limited the infiltration of MCs and activated macrophages in the colonic submucosa, although the number of eosinophils was unaffected. This was associated with a lower intestinal damage score and visceral sensitivity, suggesting that enteric glia may exert both pro-inflammatory and pro-nociceptive effects during colitis through a dynamic and selective crosstalk with the surrounding immune cells. This suggests that glia-MCs signalling is an important pathway modulating sensory and inflammatory responses during functional and organic gastrointestinal disorders [48,49]. Grubišić et al. [50] showed that deletion of connexin-43 in glia protects against the development of visceral hypersensitivity following chronic colitis by disrupting the glial-mediated activation of macrophages. Enteric glia also has immunosuppressive effects on T cells in vitro and transmit vagal anti- inflammatory signals to resident immune cells after injury [51,52]. Thus, enteric glia might support the development of visceral hyperalgesia and enhance the intestinal damage during colitis by selectively recruiting MCs and activated macrophages in the colonic submucosa.

In recent decades, enteric glia emerged as important regulators of numerous physiological processes that modulate intestinal reflexes and maintain homeostasis [53,54,55]. Enteric glia constantly monitors their extracellular environment and respond to any pathophysiological perturbation of the enteric milieu by triggering proper responses during a state-defined “reactive gliosis”. This leads to changes in the expression of receptors and gliotransmitters release with either beneficial or detrimental effects on the surrounding neuronal and non-neuronal cells. Notably, enteric glia may exhibit a pro-inflammatory phenotype that contributes to neurodegeneration and neuroplasticity during acute inflammation [2,10,56]. In several intestinal inflammatory diseases, such as Crohn’s disease, celiac disease, and ulcerative colitis in humans, enteric glia mediate NO-dependent inflammation through S100β overexpression and release [13,57]. S100β is a Ca^2+-^Zn^2+^ binding protein that is specifically expressed by enteric glia in ENS and promotes neuronal survival and neurite outgrowth at nanomolar concentration [11]. During the intestinal inflammation, S100β overexpression determines the release of pro-inflammatory cytokines via receptor for advanced glycation end (RAGE) products with mitogen-activated protein kinase (MAPK) and nuclear factor-κB (NF-κB) pathway activation [15,58,59,60]. This led to the view that S100β can be considered as an easily diffusible glial-derived pro-inflammatory cytokine that enters the extracellular space, especially at immune-inflammatory reaction sites in the gut. In line, we observed an increase in S100β expression in PLP1-positive cells within the myenteric plexuses of DNBS rats that persisted until day 7 from colitis induction. This was associated with a parallel increase in TRPV1 which was partially prevented by FC pre-treatment. The vanilloid receptor TRPV1 is one of the main nociceptive receptors, and increased TRPV1 density on immunoreactive nerve fibers was observed in the colonic mucosa of IBS patients, which correlated with the severity of perceived abdominal pain [61]. Our findings that show a parallel increase in S100β and TRPV1 within the enteric plexuses of the colon confirm the recent evidence regarding an intimate association between enteric glia and nociceptors in the intestine [10]. Gliotransmitter release is triggered by the activation of TRPV1-positive sensory neurons within the myenteric plexus, and several pro-inflammatory mediators that sensitize nociceptors also elicit responses in enteric glia [10]. This supports the role of enteric glia in contributing to nociceptor sensitization caused by inflammation, as perturbing enteric glial metabolism with FC decreases the visceromotor responses in mice [20] and visceral sensitivity in our experimental conditions. Although TRPV1 receptors expression was described in myenteric glia during the early stage of cell differentiation [62], the involvement of glial TRPV1 in visceral hyperalgesia and CNS-related pain perception remains to be investigated.

Under certain pathological circumstances, enteric glial activation is not restricted to the enteric networks and glial-dependent signaling drives neuroplastic changes throughout the gut–brain axis, leading to CNS dysfunctions [55,63,64,65]. Further, enhanced glial signaling between the intestine and the brain was incriminate in the establishment and maintenance of pain in several model of visceral hypersensitivity, suggesting the involvement of central glia [5,17,66]. We found a parallel increase in the expression of S100β and TRPV1 in DRG satellite glia and astrocytes of the PAG area after 7 days from DNBS treatment which correlated with the greater visceral sensitivity and intestinal damage. Analogously to what we observed in the colonic myenteric plexus, the expression of S100β and TRPV1 was significantly inhibited by FC, suggesting that the lack of glial activation in the intestine prevents neuroplastic changes along nociceptive pathways and persistence of abdominal pain after the resolution of acute colitis. In this context, it is important to emphasise that the effect of FC is not related to a direct inhibition of central glia since the systematic administration of FC does not affect central glia metabolism, probably because of its virtual inability to cross the blood–brain barrier [23].

Whether enteric glia have direct or indirect effects on nociceptor activity or sensitivity remains not clear, since blocking enteric glia both counteracts pain development and reduces the entity of the intestinal damage. This aspect represents a limitation of the present work that needs to be better investigated, together with the possibility that intraperitoneally administered FC might reach the DRGs and directly inhibit satellite cells. To this end, it might be important to investigate the time course of glial cell activation along the gut–brain axis during colitis.

## 5. Conclusions

In conclusion, intestinal inflammation is associated with an overexpression of TRPV1 and S100β in enteric glia and astrocytes along the gut–brain axis, which is involved in pain establishment. Our findings support the involvement of enteric glia in orchestrating pro-inflammatory and pro-nociceptive events, thereby emerging as a promising target in the attempt to reduce visceral pain induced by colitis.

## Figures and Tables

**Table 1:** Antigens and antibodies used in study.

**Primary Antibodies (Antigen)**	**Host**	**Dilution**	**Source**	**Cat#/RRID**
Tryptase	Rabbit	1:200	Genetex	GTX32931
PLP1	Mouse	1:100	Invitrogen—Thermofisher scientific	MA1-80034
TRPV1	Rabbit	1:100	Bioss	bs-1931R
S100β	Goat	1:100	Neuromics	GT15160
**Secondary antibodies**	**Host**	**Dilution**	**Source**	**Cat#/RRID**
Anti-Rabbit IgG (H + L) Secondary antibody [FITC]	Donkey	1:1000	Novusbio	NB120-6798
Anti-Mouse IgG (H-+ L) Secondary antibody [Texas Red]	Goat	1:500	Novusbio	NB120-6787
Anti-Goat IgG (H-+-L) Highly Cross-Adsorbed Secondary Antibody, Alexa Fluor Plus 647	Donkey	1:500	Invitrogen—Thermofisher Scientific	A32849

## Data Availability

The data presented in this study are available on request from the corresponding author. The data are not publicly available due to privacy restrictions.
